# An exosomes-related lncRNA prognostic model correlates with the immune microenvironment and therapy response in lung adenocarcinoma

**DOI:** 10.1007/s10238-024-01319-x

**Published:** 2024-05-18

**Authors:** Daifang Chu, Liulin Chen, Wangping Li, Haitao Zhang

**Affiliations:** Department of Respiratory and Critical Care Medicine, Tangdu Hospital, Air Force Military Medical University, 569 Xinsi Road, Xi’an, 710038 Shaanxi China

**Keywords:** Lung adenocarcinoma, Exosome, Long non-coding RNA, Tumor immune microenvironment, Prognostic model

## Abstract

Recent research highlights the significance of exosomes and long noncoding RNAs (lncRNAs) in cancer progression and drug resistance, but their role in lung adenocarcinoma (LUAD) is not fully understood. We analyzed 121 exosome-related (ER) mRNAs from the ExoBCD database, along with mRNA and lncRNA expression profiles of TCGA-LUAD using “DESeq2”, “survival,” “ConsensusClusterPlus,” “GSVA,” “estimate,” “glmnet,” “clusterProfiler,” “rms,” and “pRRophetic” R packages. This comprehensive approach included univariate cox regression, unsupervised consensus clustering, GSEA, functional enrichment analysis, and prognostic model construction. Our study identified 134 differentially expressed ER-lncRNAs, with 19 linked to LUAD prognosis. These ER-lncRNAs delineated two patient subtypes, one with poorer outcomes. Additionally, 286 differentially expressed genes were related to these ER-lncRNAs, 261 of which also correlated with LUAD prognosis. We constructed an ER-lncRNA-related prognostic model and calculated an ER-lncRNA-related risk score (ERS), revealing that a higher ERS correlates with poor overall survival in both the Meta cohort and two validation cohorts. The ERS potentially serves as an independent prognostic factor, and the prognostic model demonstrates superior predictive power. Notably, significant differences in the immune landscape were observed between the high- and low-ERS groups. Drug sensitivity analysis indicated varying responses to common chemotherapy drugs based on ERS stratification, with the high-ERS group showing greater sensitivity, except to rapamycin and erlotinib. Experimental validation confirmed that thymidine kinase 1 enhances lung cancer invasion, metastasis, and cell cycle progression. Our study pioneers an ER-lncRNA-related prognostic model for LUAD, proposing that ERS-based risk stratification could inform personalized treatment strategies to improve patient outcomes.

**Supplementary Information:**

The online version contains supplementary material available at 10.1007/s10238-024-01319-x.

## Introduction

Lung cancer is the most prevalent cancer and the major cancer-related cause of death [1], with lung adenocarcinoma (LUAD) being the most common histological type, accounting for 40% of lung cancers [2]. The incidence and mortality of LUAD are increasing year by year. In recent decades, great advances have been made in treatment strategies, such as surgery, chemotherapy, immunotherapy, radiotherapy or targeted therapy, but the 5-year survival rate for LUAD remains less than 20% [3–5]. In addition, due to the heterogeneity of tumors, criteria for identification of high-ERS patients and selection of precise treatment options are still lacking. Therefore, the exploration of robust predictor is crucial for better individualized precision medicine.

Exosomes, tiny extracellular vesicles of 30–100 nm, have been shown to play a pivotal function in intercellular communication through cell-to-cell transfer of nucleic acids and proteins, participating in a wide range of physiological processes [6, 7]. Recently, studies have revealed that exosomes also have a critical effect in tumorigenesis and progression [8, 9]. In particular, the role of exosomes in LUAD has been preliminarily elucidated, involving in LUAD progression [10, 11] and metastasis [12], drug resistance [13].

Long non-coding RNAs (lncRNAs), no less than 200 nucleotides in length, can regulate gene expression levels but are not involved in encoding proteins and play a regulatory role in physiology and disease development [14]. Evidence has suggested a potential role for lncRNAs in the onset and progression of LUAD [15]. Recent studies have revealed that lncRNAs can be encapsulated into exosomes and widely participate in intercellular signaling, which has important pathophysiological significance [16]. The exosomal lncRNAs can regulate antigen presentation, change the tumor microenvironment (TME) and participate in critical oncological behaviors, including tumor growth, metastasis, invasion [17–19]. In addition, studies have demonstrated that the dysregulation of exosomal lncRNAs are participating in multiple processes of cancer progression, making them diagnostic biomarkers and potential targets for cancer therapy [20].

However, there are few studies that focused on the role of exosomal lncRNAs in LUAD. In this study, we recognized the prognostic exosome-related lncRNAs (ER-lncRNAs) in LUAD and constructed an ER-lncRNAs signature to predict the prognosis and therapeutic response of LUAD patients. Our study may provide new directions for prognostic assessment and personalized therapy strategies for patients with LUAD.

## Materials and methods

### Data collection and preprocessing

Gene expression data (raw counts) and clinical data of the Cancer Genome Atlas (TCGA)-LUAD cohort were obtained from TCGA database (https://portal.gdc.cancer.gov/repository). Raw counts were transformed into transcripts per million (TPM) values for subsequent analysis. Gene expression data and clinical data of five independent Gene Expression Omnibus (GEO) datasets, namely, GSE30219 (*n* = 83), GSE3141 (*n* = 58), GSE50081 (*n* = 127), GSE31210 (*n* = 226) and GSE72094 (*n* = 398) were obtained from the GEO database (https://www.ncbi.nlm.nih.gov/). The ComBat algorithm in the “sva” R package was used for eliminating the batch effects between the TCGA-LUAD and GEO cohorts [21, 22]. The TCGA-LUAD, GSE30219 (*n* = 83), GSE3141 (*n* = 58), and GSE50081 (*n* = 127) cohorts were consolidated into a Meta cohort. Then, the gene expression data of Meta cohort were normalized using scale function of "limma" R package. GSE31210 (*n* = 226) and GSE72094 (*n* = 398) were used as the external validation cohorts. Besides, 121 exosome-related mRNAs (ER-mRNAs) [23] were collected from ExoBCD database (https://exobcd.liumwei.org/).

### ER-lncRNAs

112 ER-mRNAs were matched in the Meta cohort, which were used to identify ER-lncRNAs. Differentially expressed (DE) analyses of ER-mRNAs and lncRNAs were performed using the “DESeq2” R package based on raw counts data. |logFC| >1 and FDR <0.05 were set as the screening threshold. The correlations between the expression levels of DE-ER-mRNAs and DE-lncRNAs by Spearman correlation analysis, and |cor| > 0.3 and *p* < 0.05 were screened as ER-lncRNAs.

### Consensus clustering

Prognostic ER-lncRNAs were screened using univariate cox regression analysis by “survival” R package (*p* < 0.05). Subsequently, based on the expression of the prognostic ER-lncRNAs, unsupervised consensus clustering was performed to clustering patients by “ConsensusClusterPlus” R package [24]. Survival curves of the overall survival (OS) between different clusters were constructed by the Kaplan–Meier analysis. The principal component analysis (PCA) was applied to verify its validity.

### Gene set enrichment analysis (GSEA)

GSEA was performed to assess biological progress between distinct groups using the GSEA software (3.0). Gene expression data in the Meta cohort were used for GSEA analysis. The reference gene set of c2.cp.kegg.v7.0.symbols.gmt or h.all.v7.4.symbols.gmt was obtained from Molecular Signatures Database [25] (http://www.gsea-msigdb.org/gsea/downloads.jsp). The statistically significant pathways were screened with *p* < 0.05.

### Assessment of the immune landscape

The activity of immune cell, function, and pathway of each LUAD sample were calculated with a single sample gene set enrichment analysis (ssGSEA) algorithm using “GSVA” R package [26]. The “estimate” R package was used to assess the immune score, stromal score of each LUAD sample [27].

### Construction and validation of an ER-lncRNA-related prognostic model

The differentially expressed genes (DEGs) between distinct clusters were recognized using the “limma” R package with |logFC|> 1 and a false discovery rate (FDR) < 0.05. The prognostic DEGs were identifed using univariable cox regression (*p* value < 0.05), which were extracted for constructed an ER-lncRNA-related prognostic model using least absolute shrinkage and selection operator (LASSO) Cox regression analysis by “glmnet” R package [28, 29]. Based on the constructed prognostic model, the risk score for each LUAD sample in Meta cohort, which we termed as ERS, was calculated as follows: ERS = *n*∑Coefi × Expi, with *n*, Coefi and Expi representing the number of model genes, gene *i* beta coefficient value and gene expression level, respectively. Patients were divided into high- and low- ERS groups by the median of the ERS. Kaplan–Meier curve and time‐dependent receiver operating characteristic (ROC) curve by the “survival” and “timeROC” R package were used to assess the OS difference and predictive accuracy of the ERS, respectively. The univariate and multivariate Cox regression analyses were applied to evaluate the independent prognostic value of the ERS. Two separate cohorts (GSE31210, GSE72094) were used to validate the ERS. Specifically, we used the same model genes to calculate the ERS in these two validation cohorts. Patients in these two validation cohorts were also classified into high and low ERS groups, respectively, based on the median ERS values of the Meta cohort. Similarly, we also used Kaplan–Meier curves and time-dependent ROC curves to assess OS differences and predictive accuracy of ERS, respectively. Univariate and multivariate Cox regression analyses were applied to assess the independent prognostic value of ERS in the two validation cohorts.

### Functional enrichment analysis

Gene Ontology (GO) and Kyoto Encyclopedia of Genes and Genomes (KEGG) analysis were employed to explore the enrichment in biological function and pathway of the DEGs using the “clusterProfiler” R package [30, 31]. *p* values < 0.05 were statistically significant.

### Development and verification of nomogram

We established the nomogram in the Meta cohort using the constructed prognostic model and the above-collected clinicopathological parameter to anticipate the prognosis of LUAD patients by the ‘rms’ R package. Calibration curves were used to evaluate the fitness between actual and predicted survival statuses with the established nomogram. Decision curve analysis (DCA) and ROC curves were applied to identify whether this nomogram model was suitable for clinical utility using the “rms”, “rmda” and “timeROC” R packages [32–34].

### Drug sensitivity

The “pRRophetic” R package [35] was used to calculate the half-maximum inhibitory concentration (IC50) for eight common chemotherapy drugs in LUAD, including cisplatin, docetaxel, methotrexate, paclitaxel, erlotinib, gemcitabine, rapamycin and erlotinib, to predict the chemotherapy sensitivity of each LUAD sample based on the Genomics of Drug Sensitivity in Cancer database [36] (GDSC, https://www.cancerrxgene.org/).

### Cell lines, RNA interfering

NCI-H1975 and A549 Cells were cultured in Dulbecco’s Modified Eagle Medium (DMEM) culture medium, supplemented with 10% fetal bovine serum (FBS) in a standard humidified incubator with 5% CO_2_ at 37 °C. The knockdown of thymidine kinase 1 (TK1) in LUAD cells was achieved via the transfection of the TK1 specific small Interfering RNA (siRNA) using Lipofectamine 3000 reagent (Invitrogen, Massachusetts, USA) according to the manufacturer’s protocol. The TK1 specific siRNA were synthesized from GenePharma (Shanghai, China) and the sequence of siRNA is as the following: 5′-AGAAACUCAGCAGUGAAAGCC-3′. The knockdown efficiency was evaluated by Real-Time quantitative PCR (RT-qPCR) after 48 h transfection.

### Migration, invasion, and proliferation assay

Colony formation assays were employed to determine cell viability. Cells were seeded at a density of 6 × 10^2^ cells/plate in 6-well culture plates. After transduction with TK1-specific siRNA or random control, cells were cultured in normal medium for 10 days. Surviving tumor cells were fixed with 4% paraformaldehyde and stained with crystal violet before counting the colonies.

For wound healing assays, cells (1 × 10^5^ cells/well) were seeded into 6-well plates. After the formation of an adherent confluent cell monolayer, cancer cells were starved for 8 h. Wounds were created using a 10 μL pipette, photographed at 0 h, and then again after 24 h. ImageJ was used to calculate the percentage of areas covered by migrated cells.

For in vitro migration and invasion assays, 24-well transwell chambers (Transwell, Corning Costar) were utilized. In migration assays, 4 × 10^4^ cells suspended in serum-free medium were added to the upper chamber, while the lower chamber contained medium with 20% FBS. In invasion assays, the upper membranes were pre-coated with 40 μL matrigel (Matrigel™ GFR Membrane Matrix, #356231, Corning, USA), and 8 × 10^4^ cells were seeded to the upper chamber. After 24 h of culture, cells in the upper chamber were carefully cleaned with a cotton swab. Cells attached to the filter's lower surface were fixed with 4% paraformaldehyde and stained with crystal violet. The cells on the lower surface of the membrane filter were captured on camera under a microscope.

Additionally, western blot (WB) was used to detect cell cycle-related proteins. Radio-immunoprecipitation assay (RIPA) buffer was used to prepare whole cell lysates, and proteins (15 μg/well) were separated with sodium dodecyl sulfate–polyacrylamide gel electrophoresis (SDS-PAGE) gel (10%) before being transferred to polyvinylidene fluoride (PVDF) membranes. The membranes were incubated overnight with corresponding primary antibodies. Primary antibodies against CDK4 (1:1000, #12790), cyclinD (1:1000, #2978), cyclinE (1:1000, #4129), c-Myc (1:1000, #18583) and beta-Actin (1:1000, #4970) were purchased from Cell Signaling Technology (Danvers, MA, USA). Subsequently, HRP-conjugated secondary antibodies (ab6721, ab205719, Abcam) were incubated, and chemiluminescent signals were detected using enhanced chemiluminescence (ECL) solution.

### Statistical analysis

Categorical data were compared using the chi-square test, and continuous variables were compared using the Mann–Whitney test or Kruskal–Wallis test, as appropriate. All *p* values of statistical data were based on two-sided statistical tests. *p* < 0.05 was considered to be statistically significant. All the statistical analysis was conducted by the R software (version: 4.1.0).

## Results

### Identification of ER-lncRNAs

**Fig. 1:**
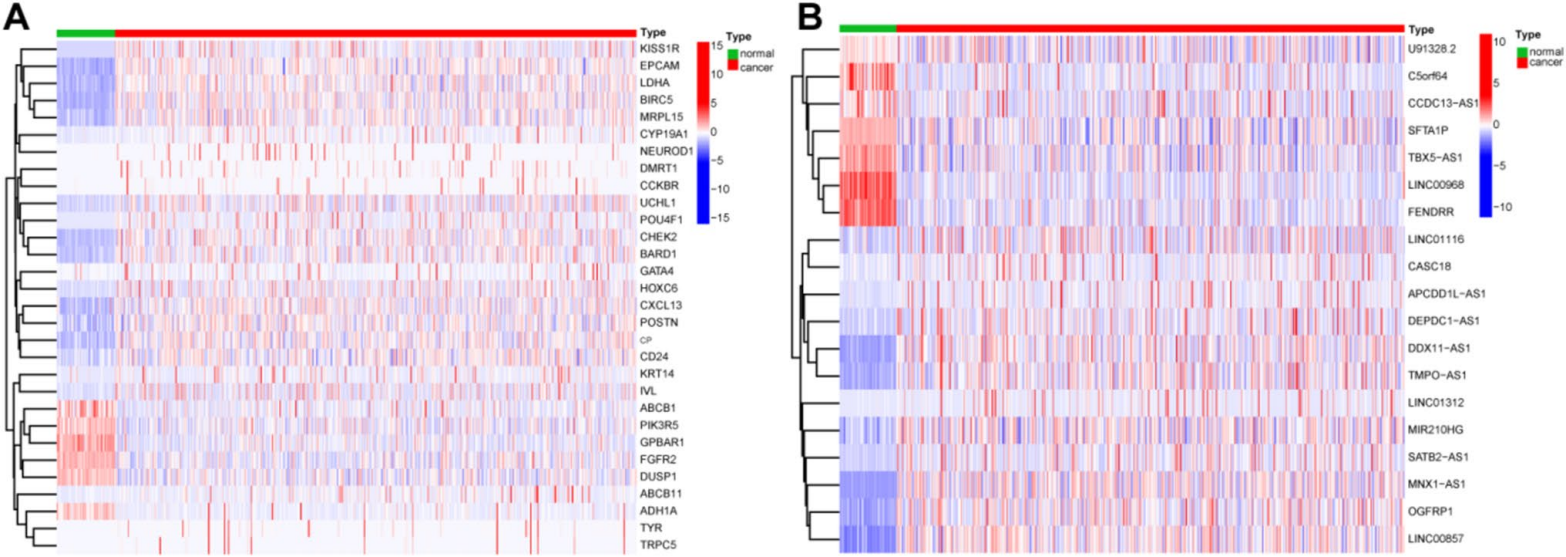
Selection of ER-lncRNAs. **A** Heatmap of differentially expressed ER-mRNAs between tumor tissues and normal tissues. **B** Heatmap of differentially expressed ER-lncRNAs between tumor tissues and normal tissues

We identified 30 DE-ER-mRNAs (Fig. 1A) and 437 DE-lncRNAs in tumor and normal patients in the TCGA-LUAD cohort. Subsequently, spearman correlation analysis was conducted on DE-ER-mRNAs and DE-lncRNAs in tumor patients, and 134 ER-lncRNAs were identified. Then, univariate COX analysis defined 19 as prognostic ER-lncRNAs (Table S1). Figure 1B shows the 19 lncRNAs expression level in tumor and normal patients.

### Identification of molecular subtypes based on prognostic ER-lncRNAs

**Fig. 2:**
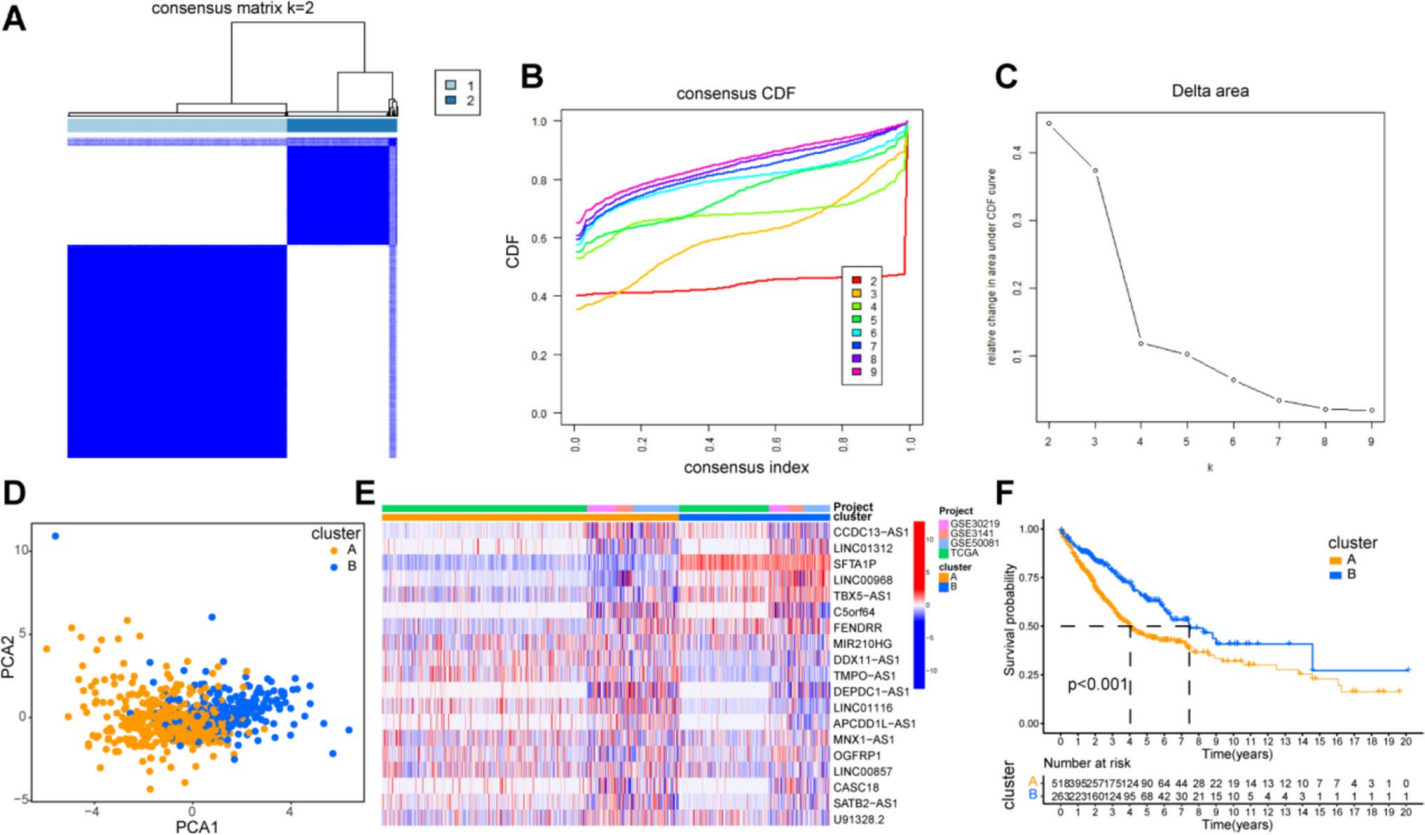
ER-lncRNA subtypes. **A** Consensus clustering matrix when *k* = 2. **B** Consensus clustering CDF with *k* valued 2–9. **C** Relative change in area under the CDF curve for *k* = 2 through 9. **D** PCA analysis showed that LUAD could be well differentiated into two subtypes based on the expression of ER-lncRNA. **E** Heatmap of the 19 prognostic ER-lncRNAs between cluster A and cluster B. **F** Kaplan–Meier curves of OS for two subtypes in LUAD

Based on 19 prognostic ER-lncRNAs, patients in Meta cohort were divided into two clusters with good internal consistency and stability (Figs. 2A–C). We termed them cluster A and B. PCA analysis showed significant difference in the two clusters (Fig. 2D). Figure 2E shows the expression of 19 prognostic ER-lncRNAs between cluster A and cluster B. Survival analysis demostrated that the prognosis of patients in cluster A was significantly worse than those in cluster B (Fig. 2F).

### Immune characterization between different ER-lncRNA-related subtypes and drug sensitivity analysis

**Fig. 3:**
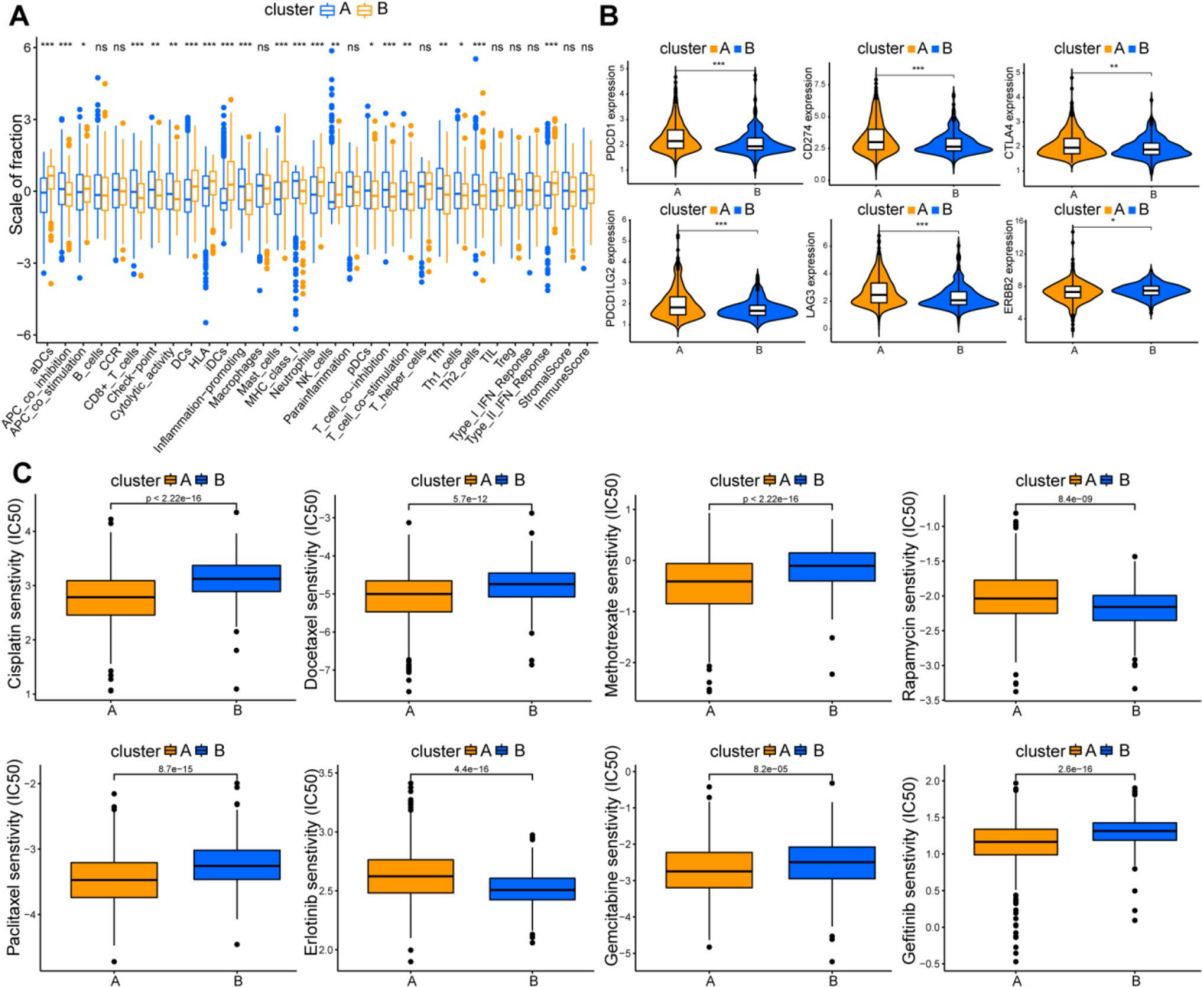
Immune characterization analysis and drug sensitivity analysis of ER-lncRNA-related subtypes. **A** The expression of immune function between different subtypes. **B** The expression levels of immune checkpoint genes between different subtypes. **C** The boxplot of sensitivity of common chemotherapy drugs in different subtypes. ns, not significant, *p < 0.05, **p < 0.01, ***p < 0.001

There are significant differences in several immune cells, functions and pathways between cluster A and cluster B (Fig. 3A). While the two clusters had no significant differences in stromal and immune scores (Fig. 3A). In addition, we investigated the expression levels of six immune checkpoint genes (i.e., PDCD1, CD274, CTLA4, PDCD1LAG2, LAG3 and ERBB2) and discovered that most of them were expressed at higher levels in cluster A than in cluster B, while ERBB2 was expressed at higher levels in cluster B (Fig. 3B). Subsequently, we explored the sensitivity of classical chemotherapy drugs in Meta cohort patients. As the IC50 value indicated, the patients in the cluster A were more sensitive to cisplatin, docetaxel, methotrexate, paclitaxel, erlotinib, and gemcitabine, while the patients in the cluster B were more sensitive to rapamycin and erlotinib (Fig. 3C).

### Biological characteristics of ER-lncRNA-related subtypes

**Fig. 4:**
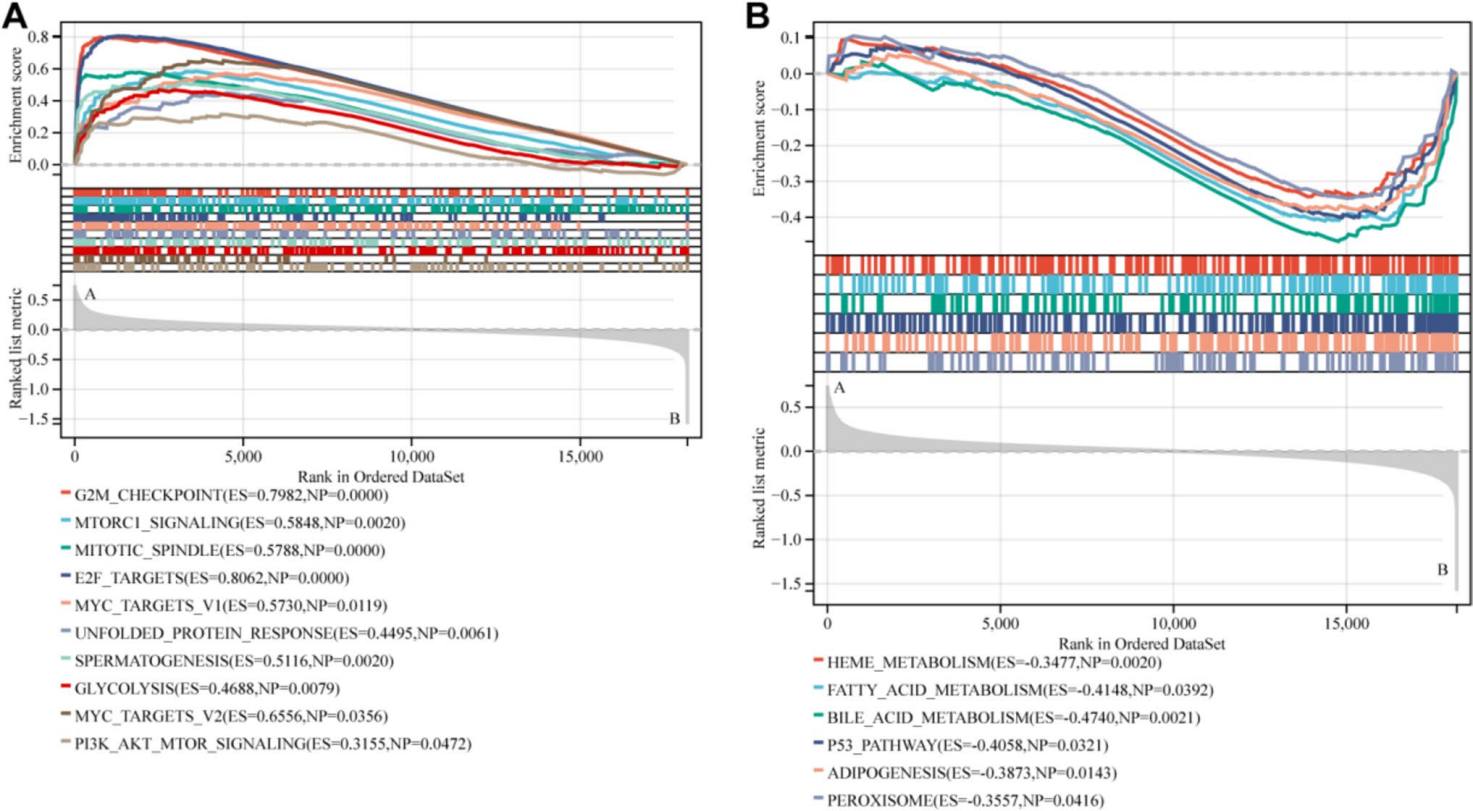
Biological characterization of ER-lncRNA-related subtypes. The GSEA pathway enrichment analysis in cluster A **A** and cluster B (**B**)

To explore the reasons for the different prognosis and immune characteristics, we investigated the hallmark pathways in cluster A and B. The GSEA results revealed that cluster A were enriched in DNA replication and the cell cycle related pathways, such as E2F target, G2M checkpoint, MYC target, MTORC1, and PI3K-Akt pathway (Fig. 4A). Cluster B has more metabolism processes, such as heme metabolism, bile acid metabolism, and fatty acid metabolism (Fig. 4B).

## Establishment and verification of an ER-lncRNA‐related prognostic model in LUAD

### Identification of the DEGs and functional annotation

**Fig. 5:**
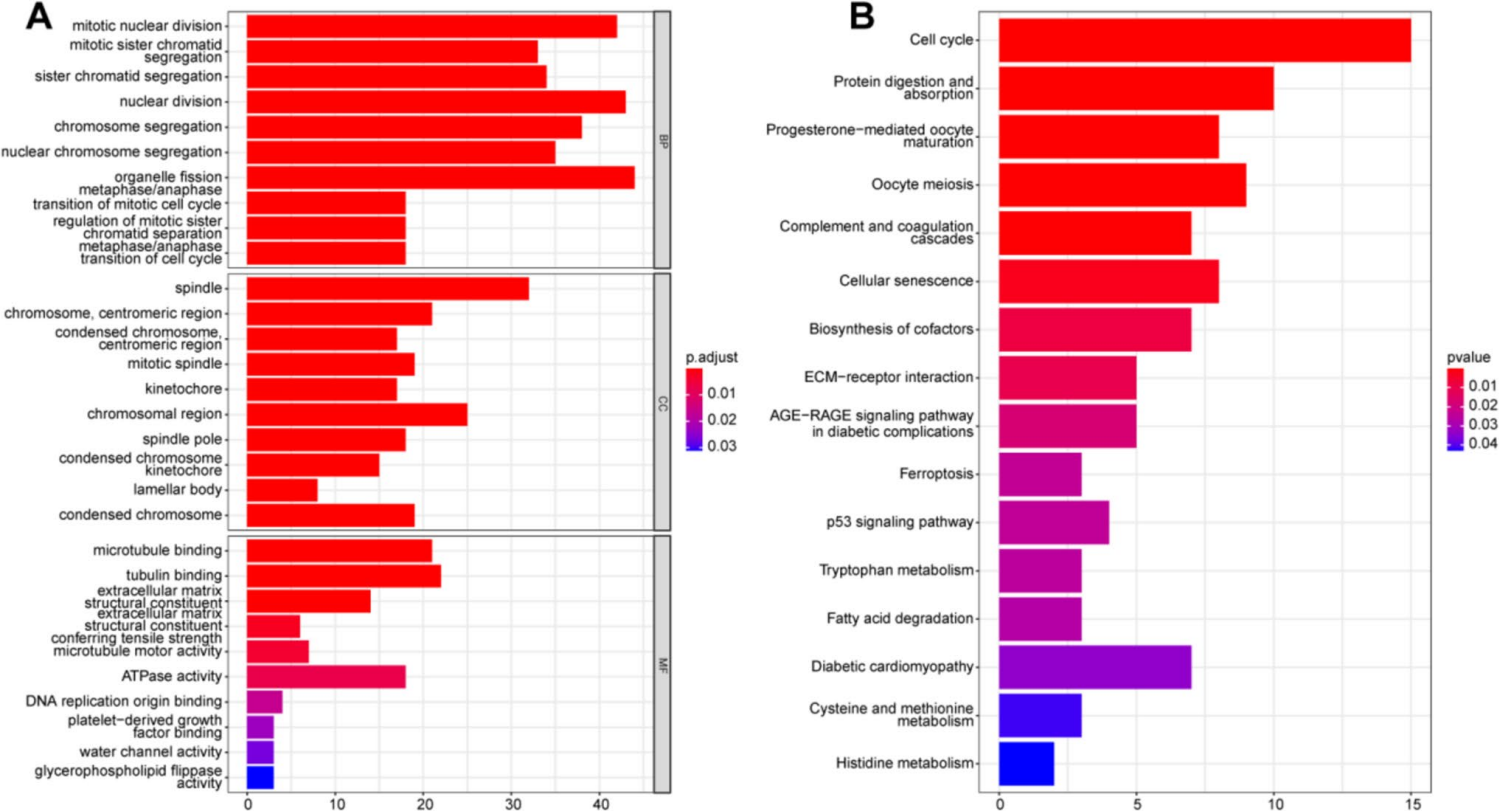
Enrichment analysis of DEGs between the two ER-lncRNA-related subtypes. Gene ontology (GO) **A** analysis of the 261 prognostic DEGs. BP, biological process; CC, cellular component; MF, molecular function. **B** Kyoto Encyclopedia of Genes and Genomes (KEGG) pathways of the 261 prognostic DEGs. *p* < 0.05

To further explore the potential biological activity of ER-lncRNA modification subtypes, we recognized 286 ER-lncRNA-subtypes-related DEGs (Table S2), 261 of which were significantly relevant to the OS of LUAD (Table S3). GO and KEGG results indicated that the 261 prognostic genes were primarily concentrated in cell cycle, metabolism and cancer-related pathways (Fig. 5).

### Establishment of ER-lncRNA‐related prognostic model

To accurately predict the ER-lncRNA-related subtype in each LUAD patients, we established a prognostic model based on the identified 261 prognostic ER-lncRNA‐related genes. Then, 13 out of the 261 prognostic ER-lncRNA‐related genes were selected as the most potent prognostic markers using LASSO Cox regression analysis. The formula for calculating the risk score is as follows: ERS = PRC1 × 0.005 + ANLN × 0.044 + SCN7A × (− 0.017) + MAMDC2 × (− 0.008) + TK1 × (0.003) + VIPR1 × (− 0.019) + CHRDL1 × (− 0.018) + GAPDH × 0.043 + LOXL2 × 0.115 + IRX3 × (− 0.015) + CPA3 × (− 0.019) + CXCL17 × (− 0.037) + FAM83A × 0.038. LUAD patients were divided into high- and low- ERS groups based on a median ERS of 0.821.

### Evaluation and validation of ER-lncRNA‐related prognostic model

**Fig. 6:**
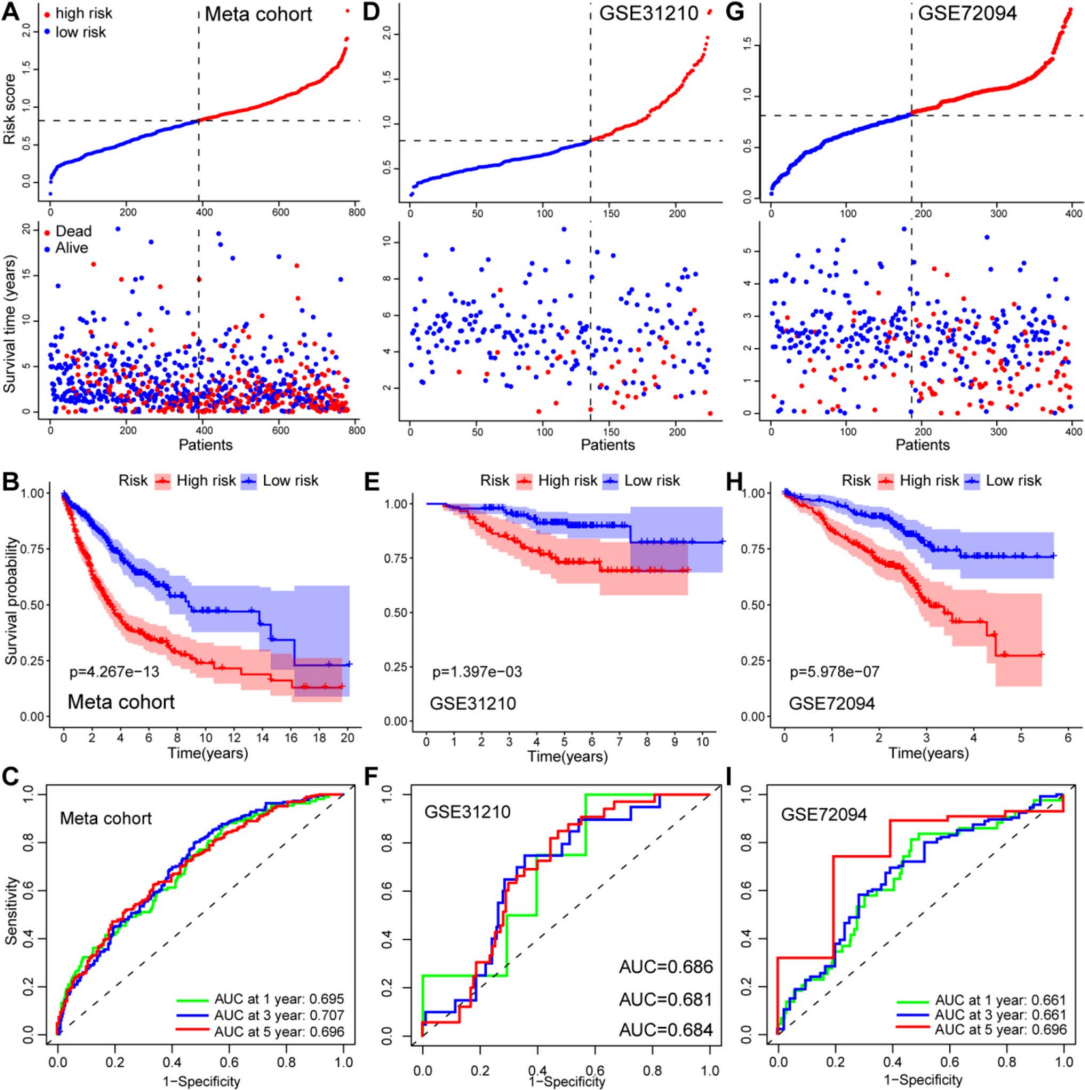
Evaluation and validation of ER-lncRNA‐related prognostic model. Distribution of ERS and survival time, Kaplan–Meier curves of overall survival and ROC curves in TCGA-LUAD (A-C), GSE31210 (**B**–**F**) and GSE72094 (**G**–**I**) cohorts

Figure 6A shows the ERS and survival time for each patient in the Meta cohort. The Kaplan–Meier curve indicated that patients in the high-ERS group had a significantly worse prognosis than those in the low-ERS group (Fig. 6B). To evaluate the validity of ERS, we established a ROC curve. The AUCs for 1, 3 and 5 years was 0.695, 0.707 and 0.696, respectively (Fig. 6C).

Furthermore, according to the same prognostic model, 227 samples from GSE31210 cohort (Fig. 6D–F) and 400 samples from GSE72094 cohort (Fig. 6G–I) were validated the superior prognostic prediction abilities of the ERS. Kaplan–Meier curves of the GSE31210 and GSE72094 validation cohorts confirmed that patients in the high-ERS group had shorter OS than those in the low-ERS group. The AUCs of the ERS in GSE31210 cohort for 1, 3 and 5 years were 0.686, 0.681 and 0.684. And the AUCs of the ERS in GSE72094 cohort for 1, 3 and 5 years were 0.661, 0.661 and 0.696. These results suggested that the prognostic model based on the 13 ER-lncRNA-related genes may be a reliable predictor of prognosis in LUAD patients.

### ERS is an independent prognostic predictor for LUAD patients

**Fig. 7:**
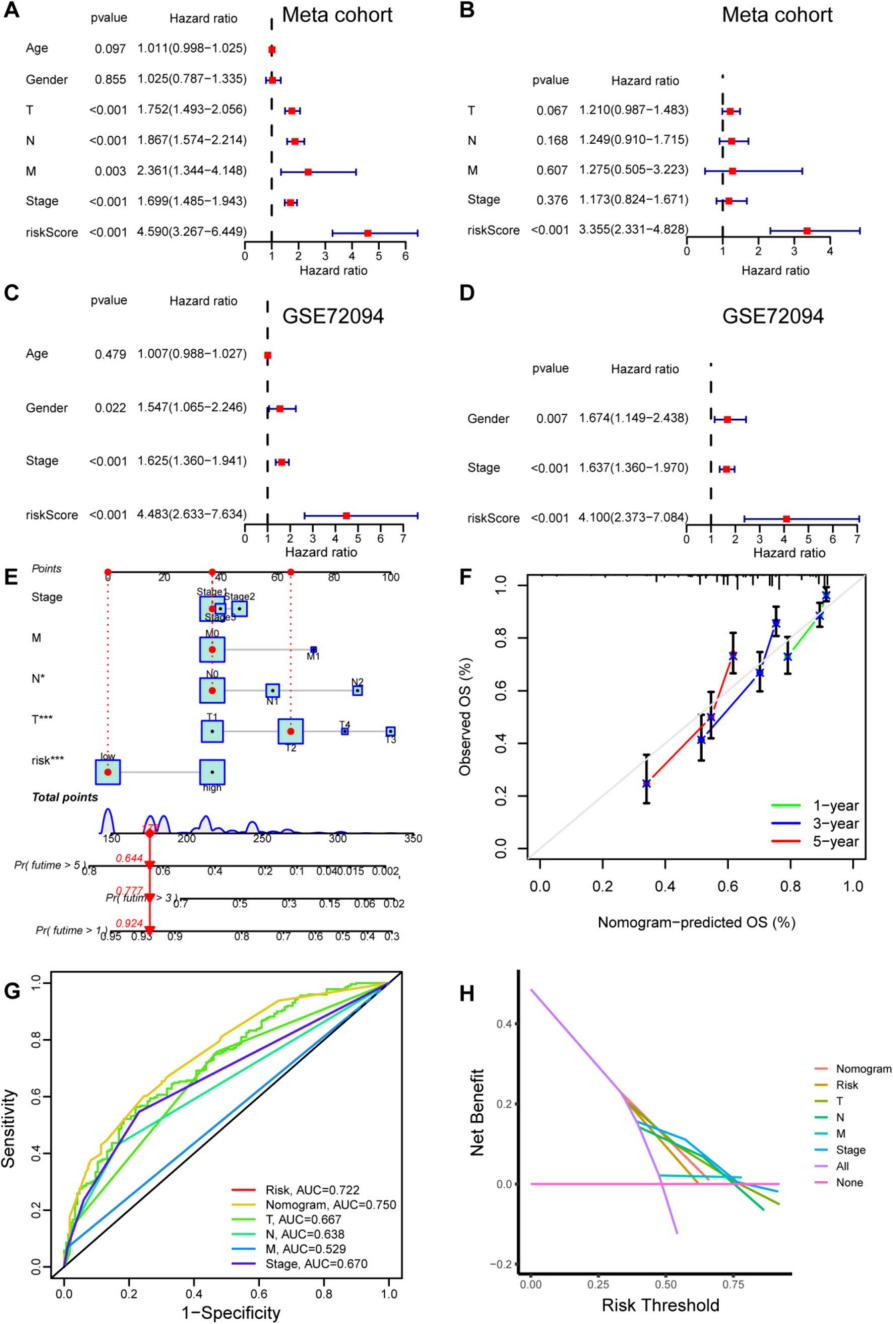
Predictive nomogram. Univariate (**A**) and multivariate **B** analysis of the clinicopathologic features and the ERS in Meta cohort. Univariate (**C**) and multivariate **D** analysis of the clinicopathologic features and the ERS in GSE72094 cohort. **E** Nomogram to predict the survival of the LUAD patients. **F** Calibration curve for 1-, 3-, and 5-year survival. ROC (**G**) and DCA **H** curve of the clinicopathologic features and risk score

Univariate cox analysis revealed that ERS, T-stage, N-stage, M-stage, and pTNM staging were significantly related to OS in patients with LUAD in the Meta cohort (Fig. 7A). Further multivariate cox analysis demonstrated that the ERS was an independent prognostic predictor with HR = 3.355 (95% CI 2.331–4.828), *p* < 0.001 (Fig. 7B). Similarly, the ERS proved to be an independent prognostic factor in GSE72094 cohort (Fig. 7C, D).

## Development and validation of the nomogram prediction model

Morever, we integrated the collected clinicopathological characteristics and the established ER-lncRNA-related prognostic model to develop an accurate prognostic nomogram model to predict the 1-, 3-, and 5- years OS of LUAD patients in the Meta cohort (Fig. 7E). The calibration curves of 1-, 3- and 5- year OS rates in the Meta cohort were close to the ideal reference line, which indicated that the nomogram model had good prediction performance (Fig. 7F). In addition, ROC and DCA results showed that the ERS and nomogram model had high reliability (Fig. 7G, H). The AUC of the 5-year OS predictions for the ERS and nomogram model were 0.722 and 0.750, respectively (Fig. 7G). The ERS and nomogram model had significantly higher benefits than extreme curves according to the DCA, and nomogram model had higher benefits than ERS (Fig. 7H).

## Biological features of low- and high- ERS group

**Fig. 8:**
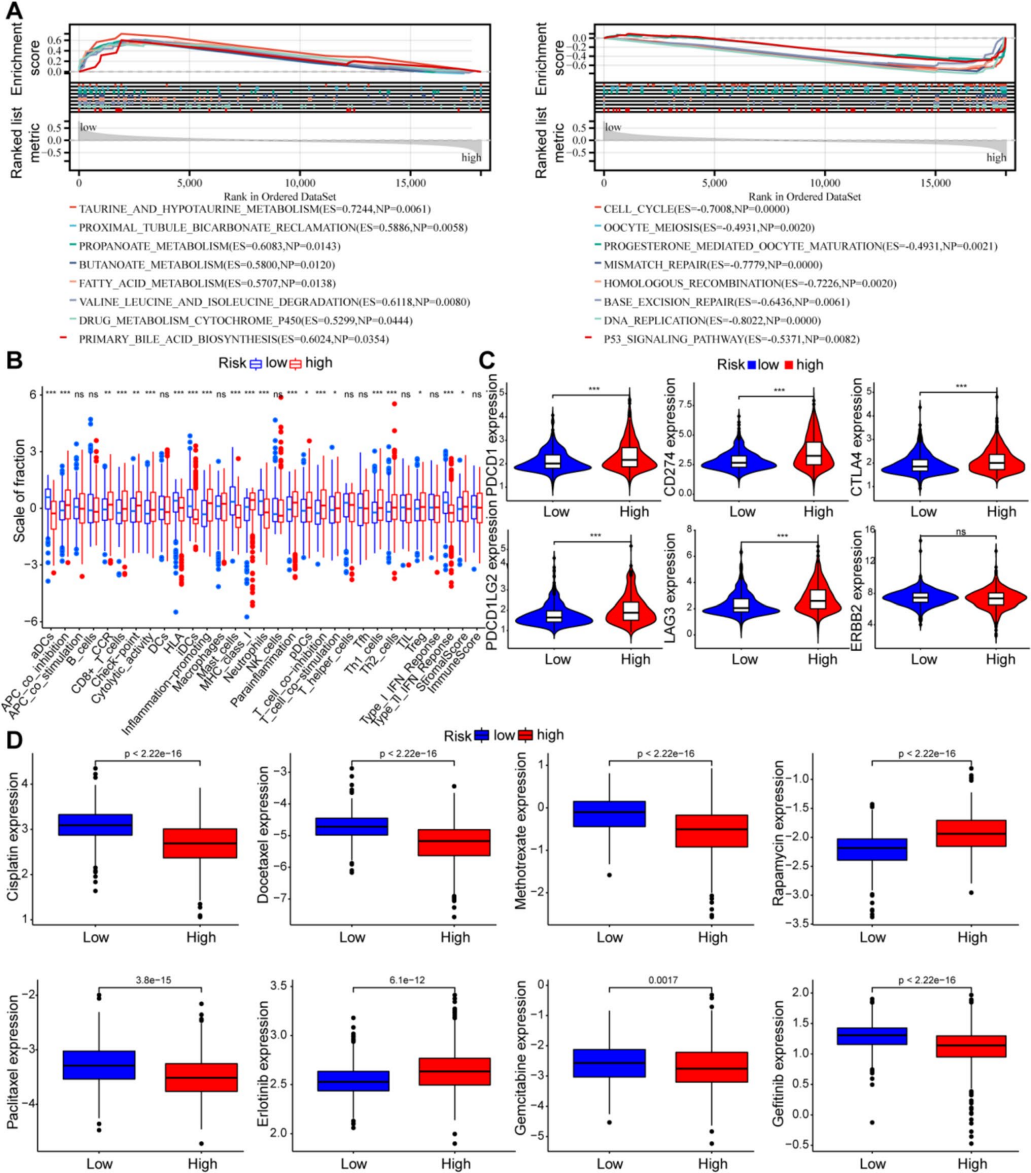
Biological characterization, immunological characterization and drug sensitivity analysis of high- and low- ERS groups. **A** The GSEA pathway enrichment analysis in low- (left) and high- (right) ERS groups. **B** The expression of immune function between different groups. **C** The expression levels of immune checkpoint genes between different groups. **D** The boxplot of sensitivity of common chemotherapy drugs in different groups. ns, not significant, *p < 0.05, **p < 0.01, ***p < 0.001

To explore the reasons for the different prognoses and immune landscapes, we investigated the hallmark pathways between the low- and high- ERS groups. The results showed that low-ERS group had more metabolism-related pathways (Fig. 8A). The high-ERS group had more cell cycle and cancer-related pathways (Fig. 8A).

## Assessment of immune characteristics and drug sensitivity in different ERS patients

There are significant differences in several immune cells, functions and pathways between low- and high- ERS groups (Fig. 8B). And the high-ERS group showed higher stromal scores than low-ERS group (Fig. 8B). In addition, except ERBB2, the immune checkpoint genes expression level (i.e., PDCD1, CD274, CTLA4, PDCD1LAG2, LAG3) were significantly higher in high-ERS group than in low-ERS group (Fig. 8C). Subsequently, the IC50 value indicated, the patients in the high-ERS group were more sensitive to classical chemotherapeutic drugs than those in low-ERS group, except for rapamycin and erlotinib (Fig. 8D).

## Knockdown of TK1 inhibited cell proliferation and metastasis of LUAD

**Fig. 9:**
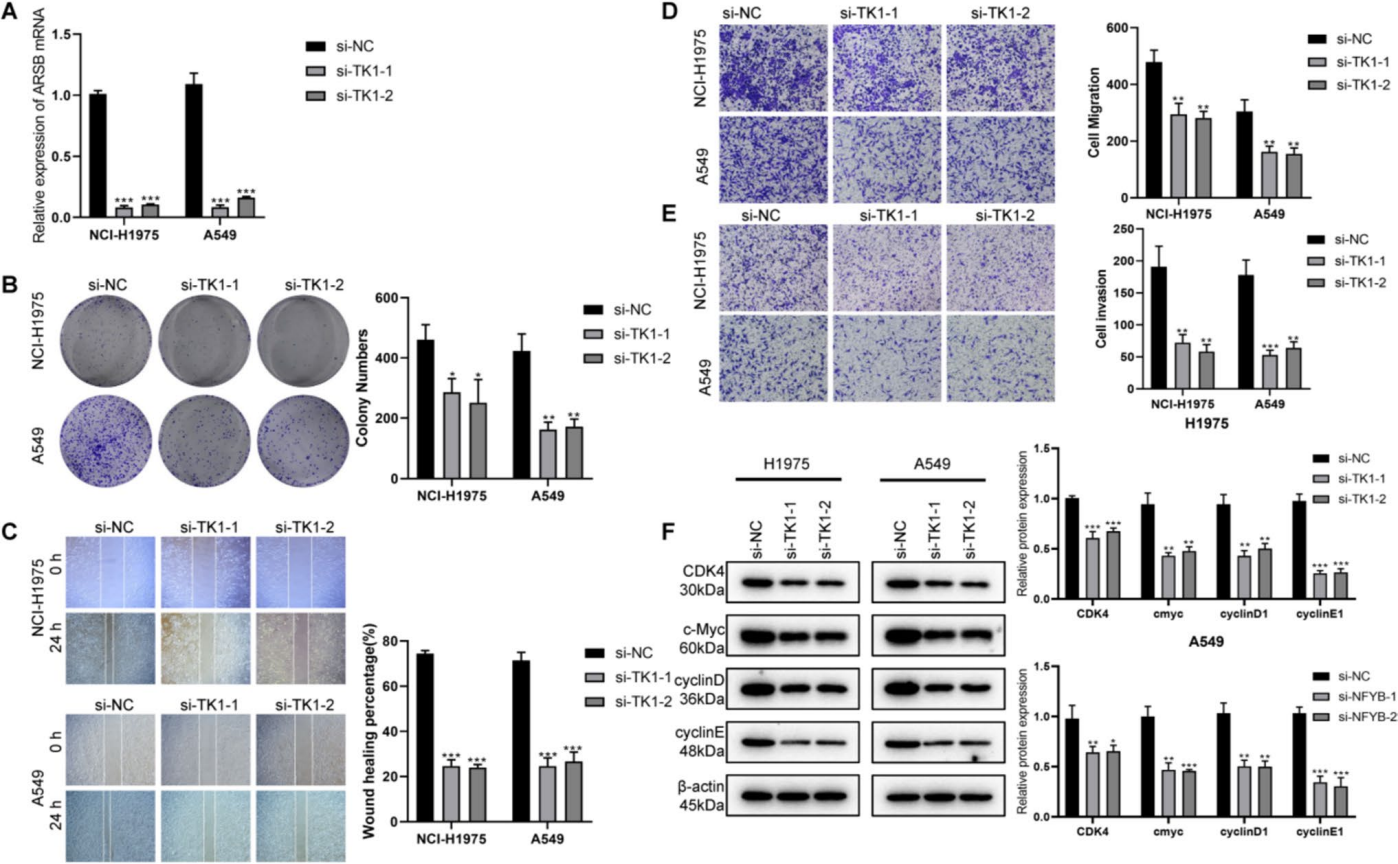
TK1 promote cell proliferation, migration, invasion and cell-cycle progression in LUAD. **A** The efficiency of TK1 silencing was confirmed by RT-qPCR in NCI-H1975 and A549 cell lines. **B** The colony assay revealed that the knockdown of TK1 significantly inhibited the proliferation ability of both NCI-H1975 and A549 cells. **C** Wound healing assays demonstrated that TK1 knockdown exerted a pronounced inhibitory effect on the migration of LUAD tumor cell lines (NCI-H1975 and A549). **D** Transwell migration assays indicated that TK1 knockdown compromised the migration ability of both NCI-H1975 and A549 cells. **E** Transwell invasion assays illustrated that TK1 knockdown attenuated the invasion ability of NCI-H1975 and A549 cells. **F** Analysis of cell cycle-related proteins in TK1 knockout cell lines revealed a significant decrease in their expression. The data presented herein are the result of at least three independent experiments

We further investigated the role of TK1 in LUAD cells, including NCI-H1975 and A549. The effectiveness of TK1 silencing in NCI-H1975 and A549 was confirmed through RT-qPCR validation (Fig. 9A). Results from colony formation assays indicated that TK1 knockdown led to a substantial suppression of cell proliferation (Fig. 9B). Subsequent wound healing assays demonstrated a significant inhibition of cell migration in NCI-H1975 and A549 upon TK1 inhibition (Fig. 9C). Moreover, the transwell migration assay illustrated that TK1 knockdown markedly diminished the migration capability of both NCI-H1975 and A549 (Fig. 9D), while the invasion ability of these cells was also compromised after TK1 knockdown, as evidenced in Fig. 9E. Additionally, there was a noteworthy reduction in the expression of cell cycle-related proteins in the TK1 knockout cell lines (Fig. 9F). Therefore, the silencing of TK1 had a substantial impact on suppressing the proliferation and metastasis of LUAD cells.

## Discussion

Currently, numerous studies have demonstrated that many exosomal lncRNAs expression levels are significantly different from normal tissues in pathological states, especially in tumor tissues. This suggests that exosomes can selectively package, translocate and secrete specific lncRNAs to regulate the corresponding biological processes [37, 38]. Exosomal lncRNAs have multiple biological functions in tumor biology, affecting tumor cell proliferation, apoptosis, migration, promoting angiogenesis, and also play an essential role in regulating the TME and mediating drug resistance [20, 39, 40]. Exosomal lncRNAs are generally considered as novel biomarkers for early diagnosis, prognosis prediction and efficacy assessment for tumors [41, 42]. Therefore, systematic studies on the prognostic implications and drug response prediction of exosomal lncRNAs in LUAD are necessary.

To our knowledge, this is the first systematic elucidation of the role of ER-lncRNAs in LUAD prognosis and drug response. We identified 134 DE-ER-lncRNAs using spearman correlation analysis, 19 of which were associated with prognosis in LUAD. Then, based on the 19 prognostic ER-lncRNAs expression, we identified two subtypes of ER-lncRNAs related in LUAD—subtype A and subtype B. Significant prognostic differences were observed between the two ER-lncRNAs-related-subtypes. GSEA results revealed that subtype A was enriched in DNA replication and cell cycle-related pathways, while subtype B had more metabolic processes. This is consistent with previous studies showing that LUAD subtypes enriched in metabolism-related pathways have a good prognosis [43]. In addition, there is a significant difference in immune cell infiltration between the two ER-lncRNAs-related-subtypes. Meanwhile, mRNA expression levels of immune checkpoints (i.e., PDCD1, CD274, CTLA4, PDCD1LAG2, LAG3, and ERBB2) were higher in subtype A than in subtype B. Consistent with this, subtype A was more sensitive to common therapeutic drugs, except rapamycin and erlotinib. Therefore, we hypothesized that ER-lncRNAs are relevant to prognosis and treatment response in LUAD patients.

Prognostic scoring systems based on gene expression have been established as robust predictors of patient outcomes [44, 45]. Although several prognostic scoring systems based on lactate metabolism-related lncRNA [46] and pyroptosis-related lncRNA [47] have been developed in LUAD, there is a scarcity of prognostic scoring systems based on the features of ER-lncRNA. To accurately predict the ER-lncRNA modification pattern in individual patients, we developed a prognostic model based on DEGs of ER-lncRNA-related-subtypes identified in the Meta cohort and well validated in two external cohorts (GSE31210 and GSE72094). The prognostic model was used to calculate the ERS of each LUAD patient. The low-ERS patients showed better OS. Moreover, ERS was shown to be an independent prognostic factor for survival of LUAD patients and had better predictive indication value compared to other common clinical biomarkers such as T/N/M-stage and pTNM staging. To explore the reasons for the prognostic differences, we performed a GSEA analysis, which showed that the low-ERS group had metabolism-related pathways, while the high-ERS group had cell cycle and cancer-related pathways.

Since TME plays a crucial role in antitumor response [48, 49], we also investigated the immune features between high- and low-ERS groups. Tumor-infiltrating immune cells, as an indicator to assess TME and treatment response [50, 51], were significantly different between the high- and low-ERS groups. In addition, we observed higher stromal scores in the high-ERS group. Studies demonstrated that higher stromal groups are involved in tumor progression by reshaping antitumor immunity and influencing the response of immunotherapy [52, 53]. Immunotherapy has made great progress in recent decades, and immune checkpoint inhibitors (ICIs) have been approved as first-line agents for patients with advanced LUAD [54], which have shown a significant survival advantage in LUAD [55, 56]. In our study, immune checkpoint genes expression levels were significantly higher in the high-ERS group than in the low-ERS group, which means that patients in the high-ERS group may benefit more from ICI compared to those in the low-ERS group. Chemotherapy remains the first line of treatment for metastatic LUAD [57]. Due to the heterogeneity of tumor, LUAD patients exhibit different sensitivities to chemotherapy. There, we evaluated the predictive value of the ERS for chemotherapy sensitivity in LUAD patients. The above results suggested that ERS can predict the effects of the chemotherapeutic and immunotherapeutic treatments and may be useful in determining the most suitable drug regimen for individual LUAD patients based on the ERS.

TK1 participates in cell proliferation and plays a potential role as a diagnostic tool and prognostic factor in evaluating cancer treatment and progression [58]. Additionally, serum levels of TK1 have been shown to be associated with cancer staging, with higher levels of TK1 indicating a more severe prognosis [58]. A clinical study indicates that elevated levels of TK1 in the serum after chemotherapy for lung cancer signify treatment failure and a poorer prognosis [59]. Our experiments also preliminarily demonstrated that silencing TK1 had a significant impact on suppressing the proliferation, metastasis, and reducing the expression of cell cycle-related proteins in LUAD cells. Further research is needed to explore whether TK1 is included in exosomes of LUAD cells.

Our study has certain limitations. Firstly, our data were obtained from an online database. While the feasibility of our risk model was demonstrated by two independent external cohorts, additional data samples may be required to further validate the results and reduce potential biases. Secondly, the online data we utilized are all based on tissue samples. Further research is needed to explore whether TK1 and other genes in the ERS model are present in exosomes or blood, and to further investigate the potential biological mechanisms underlying the relationship among ERS model genes, exosome and LUAD prognosis. Further clinical studies are needed to explore the relationship between the expression of ERS model genes in exosomes and the prognosis and treatment response of LUAD patients.

## Conclusions

We identified two ER-lncRNA-related subtypes based on the prognostic ER-lncRNAs we identified, with significant differences in clinical and immunological characteristics. In addition, based on the ER-lncRNA-related genes, we developed an ER-lncRNA-related prognostic model—ERS, and performed antitumor drug sensitivity analysis. Our findings can be used to design individualized treatment strategies for patients with different LUAD subtypes.

## Supplementary Information

Below is the link to the electronic supplementary material.

Supplementary file1 (XLS 95 KB)

## Data Availability

All the data used to support the results of this study can be found in the public databases: TCGA: https://portal.gdc.cancer.gov/repository. GEO: https://www.ncbi.nlm.nih.gov/. GDSC: https://www.cancerrxgene.org/. MSigDB: http://www.gsea-msigdb.org/gsea/downloads.jsp. ExoBCD: https://exobcd.liumwei.org/.

## References

[1] Bray F, Ferlay J, Soerjomataram I, Siegel RL, Torre LA, Jemal A. Global cancer statistics 2018: GLOBOCAN estimates of incidence and mortality worldwide for 36 cancers in 185 countries. CA Cancer J Clin. 2018;68:394–424. 10.3322/caac.21492. 30207593 10.3322/caac.21492

[2] Siegel RL, Miller KD, Jemal A. Cancer statistics, 2020. CA Cancer J Clin. 2020;70:7–30. 10.3322/caac.21590. 31912902 10.3322/caac.21590

[3] Low JL, Walsh RJ, Ang Y, Chan G, Soo RA. The evolving immuno-oncology landscape in advanced lung cancer: first-line treatment of non-small cell lung cancer. Ther Adv Med Oncol. 2019;11:1758835919870360. 10.1177/1758835919870360. 31497071 10.1177/1758835919870360 PMC6716180

[4] Li XT, Yang JJ, Wu YL, Hou J. Toward innovative combinational immunotherapy: a systems biology perspective. Cancer Treat Rev. 2018;68:1–8. 10.1016/j.ctrv.2018.05.003. 29775845 10.1016/j.ctrv.2018.05.003

[5] Lin JJ, Cardarella S, Lydon CA, Dahlberg SE, Jackman DM, Janne PA, Johnson BE. Five-year survival in EGFR-mutant metastatic lung adenocarcinoma treated with EGFR-TKIs. J Thorac Oncol. 2016;11:556–65. 10.1016/j.jtho.2015.12.103. 26724471 10.1016/j.jtho.2015.12.103 PMC4979601

[6] Kaushik S, Cuervo AM. Proteostasis and aging. Nat Med. 2015;21:1406–15. 10.1038/nm.4001. 26646497 10.1038/nm.4001

[7] Kumar P, Becker JC, Gao K, Carney RP, Lankford L, Keller BA, Herout K, Lam KS, Farmer DL, Wang A. Neuroprotective effect of placenta-derived mesenchymal stromal cells: role of exosomes. FASEB J. 2019;33:5836–49. 10.1096/fj.201800972R. 30753093 10.1096/fj.201800972R PMC6463921

[8] Zhang L, Yu D. Exosomes in cancer development, metastasis, and immunity. Biochim Biophys Acta Rev Cancer. 2019;1871:455–68. 10.1016/j.bbcan.2019.04.004. 31047959 10.1016/j.bbcan.2019.04.004 PMC6542596

[9] Thakur A, Parra DC, Motallebnejad P, Brocchi M, Chen HJ. Exosomes: small vesicles with big roles in cancer, vaccine development, and therapeutics. Bioact Mater. 2022;10:281–94. 10.1016/j.bioactmat.2021.08.029. 34901546 10.1016/j.bioactmat.2021.08.029 PMC8636666

[10] Zhou L, Lv T, Zhang Q, Zhu Q, Zhan P, Zhu S, Zhang J, Song Y. The biology, function and clinical implications of exosomes in lung cancer. Cancer Lett. 2017;407:84–92. 10.1016/j.canlet.2017.08.003. 28807820 10.1016/j.canlet.2017.08.003

[11] Wei K, Ma Z, Yang F, Zhao X, Jiang W, Pan C, Li Z, Pan X, He Z, Xu J, et al. M2 macrophage-derived exosomes promote lung adenocarcinoma progression by delivering miR-942. Cancer Lett. 2022;526:205–16. 10.1016/j.canlet.2021.10.045. 34838826 10.1016/j.canlet.2021.10.045

[12] Chen J, Zhang K, Zhi Y, Wu Y, Chen B, Bai J, Wang X. Tumor-derived exosomal miR-19b-3p facilitates M2 macrophage polarization and exosomal LINC00273 secretion to promote lung adenocarcinoma metastasis via Hippo pathway. Clin Transl Med. 2021;11: e478. 10.1002/ctm2.478. 34586722 10.1002/ctm2.478 PMC8435259

[13] Zhang K, Chen J, Li C, Yuan Y, Fang S, Liu W, Qian Y, Ma J, Chang L, Chen F, et al. Exosome-mediated transfer of SNHG7 enhances docetaxel resistance in lung adenocarcinoma. Cancer Lett. 2022;526:142–54. 10.1016/j.canlet.2021.10.029. 34715254 10.1016/j.canlet.2021.10.029

[14] Fang Y, Fullwood MJ. Roles, functions, and mechanisms of long non-coding RNAs in cancer. Genom Proteom Bioinform. 2016;14:42–54. 10.1016/j.gpb.2015.09.006. 10.1016/j.gpb.2015.09.006 PMC4792843 26883671

[15] Chen Z, Lei T, Chen X, Gu J, Huang J, Lu B, Wang Z. Long non-coding RNA in lung cancer. Clin Chim Acta. 2020;504:190–200. 10.1016/j.cca.2019.11.031. 31790697 10.1016/j.cca.2019.11.031

[16] Nie H, Liao Z, Wang Y, Zhou J, He X, Ou C. Exosomal long non-coding RNAs: emerging players in cancer metastasis and potential diagnostic biomarkers for personalized oncology. Genes Dis. 2021;8:769–80. 10.1016/j.gendis.2020.12.004. 34522707 10.1016/j.gendis.2020.12.004 PMC8427254

[17] Qian Z, Shen Q, Yang X, Qiu Y, Zhang W. The role of extracellular vesicles: an epigenetic view of the cancer microenvironment. Biomed Res Int. 2015;2015: 649161. 10.1155/2015/649161. 26582468 10.1155/2015/649161 PMC4637039

[18] Wang Z, Chen JQ, Liu JL, Tian L. Exosomes in tumor microenvironment: novel transporters and biomarkers. J Transl Med. 2016;14:297. 10.1186/s12967-016-1056-9. 27756426 10.1186/s12967-016-1056-9 PMC5070309

[19] Wu Y, Wang Y, Wei M, Han X, Xu T, Cui M. Advances in the study of exosomal lncRNAs in tumors and the selection of research methods. Biomed Pharmacother. 2020;123: 109716. 10.1016/j.biopha.2019.109716. 31896067 10.1016/j.biopha.2019.109716

[20] Wang Y, Zhang M, Zhou F. Biological functions and clinical applications of exosomal long non-coding RNAs in cancer. J Cell Mol Med. 2020;24:11656–66. 10.1111/jcmm.15873. 32924276 10.1111/jcmm.15873 PMC7578871

[21] Irizarry RA, Hobbs B, Collin F, Beazer-Barclay YD, Antonellis KJ, Scherf U, Speed TP. Exploration, normalization, and summaries of high density oligonucleotide array probe level data. Biostatistics. 2003;4:249–64. 10.1093/biostatistics/4.2.249. 12925520 10.1093/biostatistics/4.2.249

[22] Johnson WE, Li C, Rabinovic A. Adjusting batch effects in microarray expression data using empirical Bayes methods. Biostatistics. 2007;8:118–27. 10.1093/biostatistics/kxj037. 16632515 10.1093/biostatistics/kxj037

[23] Zhao F, Li Z, Dong Z, Wang Z, Guo P, Zhang D, Li S. Exploring the potential of exosome-related LncRNA pairs as predictors for immune microenvironment, survival outcome, and microbiotain landscape in esophageal squamous cell carcinoma. Front Immunol. 2022;13: 918154. 10.3389/fimmu.2022.918154. 35880180 10.3389/fimmu.2022.918154 PMC9307964

[24] Wilkerson MD, Hayes DN. ConsensusClusterPlus: a class discovery tool with confidence assessments and item tracking. Bioinformatics. 2010;26:1572–3. 10.1093/bioinformatics/btq170. 20427518 10.1093/bioinformatics/btq170 PMC2881355

[25] Subramanian A, Tamayo P, Mootha VK, Mukherjee S, Ebert BL, Gillette MA, Paulovich A, Pomeroy SL, Golub TR, Lander ES, et al. Gene set enrichment analysis: a knowledge-based approach for interpreting genome-wide expression profiles. Proc Natl Acad Sci USA. 2005;102:15545–50. 10.1073/pnas.0506580102. 16199517 10.1073/pnas.0506580102 PMC1239896

[26] Hanzelmann S, Castelo R, Guinney J. GSVA: gene set variation analysis for microarray and RNA-seq data. BMC Bioinform. 2013;14:7. 10.1186/1471-2105-14-7. 10.1186/1471-2105-14-7 PMC3618321 23323831

[27] Du J, Tao Q, Liu Y, Huang Z, Jin H, Lin W, Huang X, Zeng J, Zhao Y, Liu L, et al. Assessment of the targeted effect of Sijunzi decoction on the colorectal cancer microenvironment via the ESTIMATE algorithm. PLoS ONE. 2022;17: e0264720. 10.1371/journal.pone.0264720. 35303006 10.1371/journal.pone.0264720 PMC8932555

[28] Simon N, Friedman J, Hastie T, Tibshirani R. Regularization paths for Cox’s proportional hazards model via coordinate descent. J Stat Softw. 2011;39:1–13. 10.18637/jss.v039.i05. 27065756 10.18637/jss.v039.i05 PMC4824408

[29] Tibshirani R. The lasso method for variable selection in the Cox model. Stat Med. 1997;16:385–95. 10.1002/(sici)1097-0258(19970228)16:4%3c385::aid-sim380%3e3.0.co;2-3. 9044528 10.1002/(sici)1097-0258(19970228)16:4<385::aid-sim380>3.0.co;2-3

[30] Kanehisa M. Toward understanding the origin and evolution of cellular organisms. Protein Sci. 2019;28:1947–51. 10.1002/pro.3715. 31441146 10.1002/pro.3715 PMC6798127

[31] Kanehisa M, Furumichi M, Sato Y, Ishiguro-Watanabe M, Tanabe M. KEGG: integrating viruses and cellular organisms. Nucleic Acids Res. 2021;49:D545–51. 10.1093/nar/gkaa970. 33125081 10.1093/nar/gkaa970 PMC7779016

[32] Heagerty PJ, Zheng Y. Survival model predictive accuracy and ROC curves. Biometrics. 2005;61:92–105. 10.1111/j.0006-341X.2005.030814.x. 15737082 10.1111/j.0006-341X.2005.030814.x

[33] Tataranni T, Piccoli C. Dichloroacetate (DCA) and cancer: an overview towards clinical applications. Oxid Med Cell Longev. 2019;2019:8201079. 10.1155/2019/8201079. 31827705 10.1155/2019/8201079 PMC6885244

[34] Kerr KF, Brown MD, Zhu K, Janes H. Assessing the clinical impact of risk prediction models with decision curves: guidance for correct interpretation and appropriate use. J Clin Oncol. 2016;34:2534–40. 10.1200/JCO.2015.65.5654. 27247223 10.1200/JCO.2015.65.5654 PMC4962736

[35] Geeleher P, Cox N, Huang RS. pRRophetic: an R package for prediction of clinical chemotherapeutic response from tumor gene expression levels. PLoS ONE. 2014;9: e107468. 10.1371/journal.pone.0107468. 25229481 10.1371/journal.pone.0107468 PMC4167990

[36] Yang W, Soares J, Greninger P, Edelman EJ, Lightfoot H, Forbes S, Bindal N, Beare D, Smith JA, Thompson IR, et al. Genomics of drug sensitivity in cancer (GDSC): a resource for therapeutic biomarker discovery in cancer cells. Nucleic Acids Res. 2013;41:D955-961. 10.1093/nar/gks1111. 23180760 10.1093/nar/gks1111 PMC3531057

[37] Cheng J, Meng J, Zhu L, Peng Y. Exosomal noncoding RNAs in Glioma: biological functions and potential clinical applications. Mol Cancer. 2020;19:66. 10.1186/s12943-020-01189-3. 32213181 10.1186/s12943-020-01189-3 PMC7098115

[38] Sun Z, Yang S, Zhou Q, Wang G, Song J, Li Z, Zhang Z, Xu J, Xia K, Chang Y, et al. Emerging role of exosome-derived long non-coding RNAs in tumor microenvironment. Mol Cancer. 2018;17:82. 10.1186/s12943-018-0831-z. 29678180 10.1186/s12943-018-0831-z PMC5909226

[39] Wang M, Zhou L, Yu F, Zhang Y, Li P, Wang K. The functional roles of exosomal long non-coding RNAs in cancer. Cell Mol Life Sci. 2019;76:2059–76. 10.1007/s00018-019-03018-3. 30683984 10.1007/s00018-019-03018-3 PMC11105177

[40] Giallombardo M, Taverna S, Alessandro R, Hong D, Rolfo C. Exosome-mediated drug resistance in cancer: the near future is here. Ther Adv Med Oncol. 2016;8:320–2. 10.1177/1758834016648276. 27583023 10.1177/1758834016648276 PMC4981289

[41] Zhao R, Zhang Y, Zhang X, Yang Y, Zheng X, Li X, Liu Y, Zhang Y. Exosomal long noncoding RNA HOTTIP as potential novel diagnostic and prognostic biomarker test for gastric cancer. Mol Cancer. 2018;17:68. 10.1186/s12943-018-0817-x. 29486794 10.1186/s12943-018-0817-x PMC6389063

[42] Kok VC, Yu CC. Cancer-derived exosomes: their role in cancer biology and biomarker development. Int J Nanomed. 2020;15:8019–36. 10.2147/IJN.S272378. 10.2147/IJN.S272378 PMC7585279 33116515

[43] Yuan C, Chen H, Tu S, Huang HY, Pan Y, Gui X, Kuang M, Shen X, Zheng Q, Zhang Y, et al. A systematic dissection of the epigenomic heterogeneity of lung adenocarcinoma reveals two different subclasses with distinct prognosis and core regulatory networks. Genome Biol. 2021;22:156. 10.1186/s13059-021-02376-1. 34001209 10.1186/s13059-021-02376-1 PMC8127276

[44] Cheong JH, Wang SC, Park S, Porembka MR, Christie AL, Kim H, Kim HS, Zhu H, Hyung WJ, Noh SH, et al. Development and validation of a prognostic and predictive 32-gene signature for gastric cancer. Nat Commun. 2022;13:774. 10.1038/s41467-022-28437-y. 35140202 10.1038/s41467-022-28437-y PMC8828873

[45] Zhu L, Wang H, Jiang C, Li W, Zhai S, Cai X, Wang X, Liao L, Tao F, Jin D, et al. Clinically applicable 53-gene prognostic assay predicts chemotherapy benefit in gastric cancer: a multicenter study. EBioMedicine. 2020;61: 103023. 10.1016/j.ebiom.2020.103023. 33069062 10.1016/j.ebiom.2020.103023 PMC7569189

[46] Mai S, Liang L, Mai G, Liu X, Diao D, Cai R, Liu L. Development and validation of lactate metabolism-related lncRNA signature as a prognostic model for lung adenocarcinoma. Front Endocrinol. 2022;13: 829175. 10.3389/fendo.2022.829175. 10.3389/fendo.2022.829175 PMC9004472 35422758

[47] Song J, Sun Y, Cao H, Liu Z, Xi L, Dong C, Yang R, Shi Y. A novel pyroptosis-related lncRNA signature for prognostic prediction in patients with lung adenocarcinoma. Bioengineered. 2021;12:5932–49. 10.1080/21655979.2021.1972078. 34488540 10.1080/21655979.2021.1972078 PMC8806662

[48] Becht E, de Reynies A, Giraldo NA, Pilati C, Buttard B, Lacroix L, Selves J, Sautes-Fridman C, Laurent-Puig P, Fridman WH. Immune and stromal classification of colorectal cancer is associated with molecular subtypes and relevant for precision immunotherapy. Clin Cancer Res. 2016;22:4057–66. 10.1158/1078-0432.CCR-15-2879. 26994146 10.1158/1078-0432.CCR-15-2879

[49] Mlecnik B, Bindea G, Angell HK, Maby P, Angelova M, Tougeron D, Church SE, Lafontaine L, Fischer M, Fredriksen T, et al. Integrative analyses of colorectal cancer show immunoscore is a stronger predictor of patient survival than microsatellite instability. Immunity. 2016;44:698–711. 10.1016/j.immuni.2016.02.025. 26982367 10.1016/j.immuni.2016.02.025

[50] Bi KW, Wei XG, Qin XX, Li B. BTK has potential to be a prognostic factor for lung adenocarcinoma and an indicator for tumor microenvironment remodeling: a study based on TCGA data mining. Front Oncol. 2020;10:424. 10.3389/fonc.2020.00424. 32351880 10.3389/fonc.2020.00424 PMC7175916

[51] Gajewski TF, Schreiber H, Fu YX. Innate and adaptive immune cells in the tumor microenvironment. Nat Immunol. 2013;14:1014–22. 10.1038/ni.2703. 24048123 10.1038/ni.2703 PMC4118725

[52] Calon A, Lonardo E, Berenguer-Llergo A, Espinet E, Hernando-Momblona X, Iglesias M, Sevillano M, Palomo-Ponce S, Tauriello DV, Byrom D, et al. Stromal gene expression defines poor-prognosis subtypes in colorectal cancer. Nat Genet. 2015;47:320–9. 10.1038/ng.3225. 25706628 10.1038/ng.3225

[53] Turley SJ, Cremasco V, Astarita JL. Immunological hallmarks of stromal cells in the tumour microenvironment. Nat Rev Immunol. 2015;15:669–82. 10.1038/nri3902. 26471778 10.1038/nri3902

[54] Han RH, Dunn GP, Chheda MG, Kim AH. The impact of systemic precision medicine and immunotherapy treatments on brain metastases. Oncotarget. 2019;10:6739–53. 10.18632/oncotarget.27328. 31803366 10.18632/oncotarget.27328 PMC6877099

[55] Doroshow DB, Sanmamed MF, Hastings K, Politi K, Rimm DL, Chen L, Melero I, Schalper KA, Herbst RS. Immunotherapy in non-small cell lung cancer: facts and hopes. Clin Cancer Res. 2019;25:4592–602. 10.1158/1078-0432.CCR-18-1538. 30824587 10.1158/1078-0432.CCR-18-1538 PMC6679805

[56] Peters S, Reck M, Smit EF, Mok T, Hellmann MD. How to make the best use of immunotherapy as first-line treatment of advanced/metastatic non-small-cell lung cancer. Ann Oncol. 2019;30:884–96. 10.1093/annonc/mdz109. 30912805 10.1093/annonc/mdz109

[57] Li T, Pan K, Ellinwood AK, Cress RD. Survival trends of metastatic lung cancer in California by age at diagnosis, gender, race/ethnicity, and histology, 1990–2014. Clin Lung Cancer. 2021;22:e602–11. 10.1016/j.cllc.2020.11.005. 33414054 10.1016/j.cllc.2020.11.005 PMC8141544

[58] Bitter EE, Townsend MH, Erickson R, Allen C, O’Neill KL. Thymidine kinase 1 through the ages: a comprehensive review. Cell Biosci. 2020;10:138. 10.1186/s13578-020-00493-1. 33292474 10.1186/s13578-020-00493-1 PMC7694900

[59] Nisman B, Nechushtan H, Biran H, Gantz-Sorotsky H, Peled N, Gronowitz S, Peretz T. Serum thymidine kinase 1 activity in the prognosis and monitoring of chemotherapy in lung cancer patients: a brief report. J Thorac Oncol. 2014;9:1568–72. 10.1097/jto.0000000000000276. 25521401 10.1097/JTO.0000000000000276

